# CircRTN4 promotes pancreatic cancer progression through a novel CircRNA-miRNA-lncRNA pathway and stabilizing epithelial-mesenchymal transition protein

**DOI:** 10.1186/s12943-021-01481-w

**Published:** 2022-01-04

**Authors:** Chi Hin Wong, Ut Kei Lou, Frederic Khe-Cheong Fung, Joanna H. M. Tong, Chang-hua Zhang, Ka-Fai To, Stephen Lam Chan, Yangchao Chen

**Affiliations:** 1 School of Biomedical Sciences, Faculty of Medicine, The Chinese University of Hong Kong, Shatin, NT Hong Kong; 2 Department of Anatomical and Cellular Pathology, Prince of Wales Hospital, The Chinese University of Hong Kong, Shatin, Hong Kong; 3 Digestive Medicine Center, The Seventh Affiliated Hospital of Sun Yat-Sen University, Shenzhen, 518107 Guangdong China; 4 Department of Clinical Oncology, Prince of Wales Hospital, The Chinese University of Hong Kong, Shatin, Hong Kong; 5 Shenzhen Research Institute, The Chinese University of Hong Kong, Shenzhen, 518087 China

**Keywords:** circRTN4, Epithelial-mesenchymal transition, HOTTIP, MiRNAs, Pancreatic ductal adenocarcinoma

## Abstract

**Background:**

Circular RNAs (circRNAs) play important roles in many biological processes. However, the detailed mechanism underlying the critical roles of circRNAs in cancer remains largely unexplored. We aim to explore the molecular mechanisms of circRTN4 with critical roles in pancreatic ductal adenocarcinoma (PDAC).

**Methods:**

CircRTN4 expression level was examined in PDAC primary tumors. The oncogenic roles of circRTN4 in PDAC tumor growth and metastasis were studied in mouse tumor models. Bioinformatics analysis, luciferase assay and miRNA pulldown assay were performed to study the novel circRTN4-miRNA-lncRNA pathway. To identify circRTN4-interacting proteins, we performed circRNA-pulldown and mass spectrometry in PDAC cells. Protein stability assay and 3-Dimensional structure modeling were performed to reveal the role of circRTN4 in stabilizing RAB11FIP1.

**Results:**

CircRTN4 was significantly upregulated in primary tumors from PDAC patients. *In vitro* and *in vivo* functional studies revealed that circRTN4 promoted PDAC tumor growth and liver metastasis. Mechanistically, circRTN4 interacted with tumor suppressor miR-497-5p in PDAC cells. CircRTN4 knockdown upregulated miR-497-5p to inhibit the oncogenic lncRNA HOTTIP expression. Furthermore, we identified critical circRTN4-intercting proteins by circRNA-pulldown in PDAC cells. CircRTN4 interacted with important epithelial-mesenchymal transition (EMT)- driver RAB11FIP1 to block its ubiquitination site. We found that circRTN4 knockdown promoted the degradation of RAB11FIP1 by increasing its ubiquitination. Also, circRTN4 knockdown inhibited the expression of RAB11FIP1-regulating EMT-markers Slug, Snai1, Twist, Zeb1 and N-cadherin in PDAC.

**Conclusion:**

The upregulated circRTN4 promotes tumor growth and liver metastasis in PDAC through the novel circRTN4-miR-497-5p-HOTTIP pathway. Also, circRTN4 stabilizes RAB11FIP1 to contribute EMT.

**Graphical abstract:**

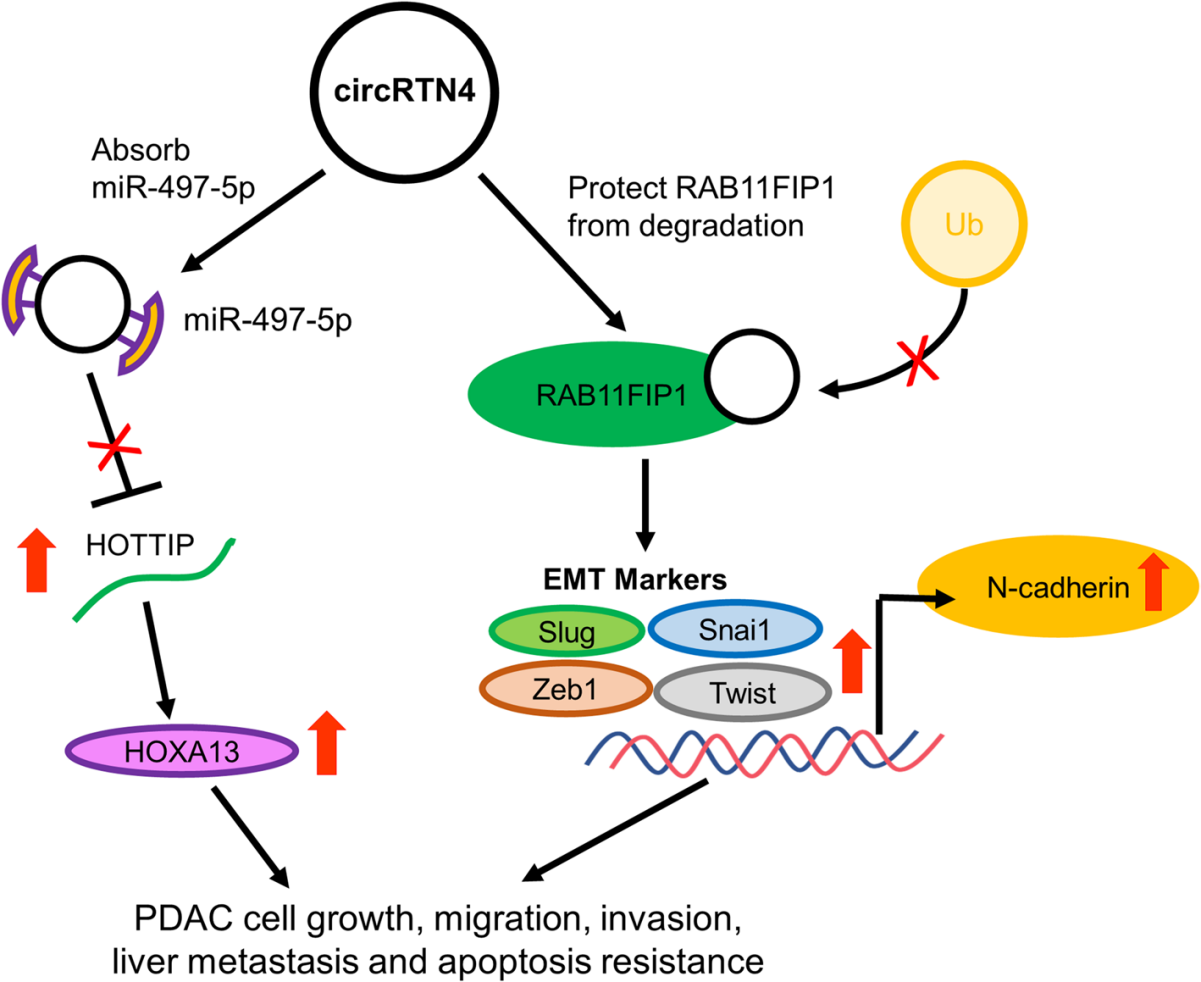

**Supplementary Information:**

The online version contains supplementary material available at 10.1186/s12943-021-01481-w.

## Introduction

Pancreatic ductal adenocarcinoma (PDAC) is one of the leading causes of cancer mortality worldwide [1]. Delayed diagnosis and ineffective treatment regimen frequently result in poor prognosis with low overall survival (9%) in PDAC patients [2–4]. Therefore, understanding the mechanism underlying PDAC initiation and progression is crucial for the development of novel diagnostic biomarkers and therapeutic targets.

Circular RNAs (circRNAs) represent a class of non-coding RNAs with the covalently joining of the 3′ end of a transcript to its 5′ end in a circular structure. Emerging studies identified important roles of circRNAs dysregulation in cancer progression [5–7]. Although circRNAs are frequently reported to regulate gene expression through functioning as microRNA (miRNA) sponges [8], many circRNAs have much lower abundance than miRNAs. This indicates that circRNAs may have additional molecular mechanisms in regulating gene expression. Particularly, the detailed functions and critical mechanisms by circRNAs in PDAC are still largely unexplored.

We profiled circRNAs expression in PDAC to identify circRNAs that played important roles in PDAC [9]. One of the upregulated circRNAs was examined in current study. circRTN4 (also named as hsa_circ_0001006), which was derived from the exon 4 and 5 of *Reticulon 4 (RTN4)* mRNA, was frequently upregulated in PDAC cells and primary tumors. We found that circRTN4 promoted PDAC cell growth, migration, and invasion. Also, *in vivo* studies demonstrated that circRTN4 promoted tumor growth and liver metastasis. Mechanistically, we identified a novel circRNA-miRNA-long non-coding RNA (lncRNA) pathway in PDAC. CircRTN4 promoted the oncogenic lncRNA HOXA Transcript At The Distal Tip (HOTTIP) expression by sponging miR-497-5p. In additional to regulating lncRNA expression, circRTN4 interacted with RAB11FIP1 to enhance its stability for promoting epithelial-mesenchymal transition (EMT) in PDAC. Collectively, our results demonstrated that circRTN4 played critical roles in PDAC progression through promoting the expression of HOTTIP and enhancing the stability RAB11FIP1 for EMT. Our results broaden the understanding on the roles of circRNAs in regulating gene expression in PDAC.

## Methods

### Clinical samples and mammalian cell lines

88 pairs of PDAC primary tumor and adjacent non-tumor tissues were obtained from patients who underwent pancreatic resection at the Prince of Wales Hospital, Hong Kong. All specimens were fixed and embedded into paraffin. HEK293 cells and PDAC cell lines PANC-1, CFPAC-1, SW1990, CAPAN-2, and BxPC-3 were obtained from American Type Culture Collection (Manassas, VA, USA). Human pancreatic ductal epithelial (HPDE) cell line was generously provided by Dr. Tsao (University Health Network, Ontario Cancer Institute and Princess Margaret Hospital Site, Toronto) [10]. All cell lines were verified by short tandem repeat profiling at the GENEWIZ, Inc. within 6 months of use, and were cultured under the condition as described previously [9].

### Plasmid and oligonucleotide transfection

CircRNA overexpression plasmid was constructed by cloning the RTN4 exon 4 and 5 into pcDNA3.1 (+) circRNA mini vector, which was a gift from Jeremy Wilusz (Addgene plasmid # 60648) [9, 11]. pmiR-circRTN4 reporter plasmid for luciferase assay was constructed by cloning circRTN4 sequence into region directly downstream of the firefly luciferase gene in the pmiR-Reporter (Promega, Madison, WI, USA). Mutation in the miRNA binding site of the pmiR-circRTN4 reporter plasmid and the RAB11FIP1 binding site of the pcDNA3.1 (+)-circRTN4 plasmid were generated using KAPA HiFi DNA Polymerase (KapaBiosystem, St. Louis, MO, USA) and primers with the mutation site. SiRNAs and miRNAs mimics were purchased from GenePharma (Shanghai, China). The sequences were presented in the Supplementary Table 1. Plasmids, siRNAs and miRNAs transfection were performed by Lipofectamine 3000 (Invitrogen, Waltham, MA, USA), according to the manufacturer’s protocol.

### Lentiviral production and infection

Construction of the lentiviral vector for shRNA knockdown and the establishment of stable knockdown CFPAC-1 cell lines were described previously [12]. Briefly, shRNA sequence targeting circRTN4 was cloned into lentiviral transfer vector. The VSV-G-pseudotyped lentivirus was produced by co-transfecting packaging vectors: pCMV-VSVG, pRSV-REV and pMDLg/pRRE with transfer vectors in HEK293T cells. CFAPC-1 cells were infected with sh-circRTN4 lentivirus with hexadimethrine bromide (Polybrene) (Sigma, St. Louis, MO, USA) for 72 h. Selection with 800 μg/ml geneticin for 2 weeks was performed for establishing the stable sh-circRTN4 clone. The efficiency of knockdown was confirmed by qRT-PCR.

### *In vivo* PDAC mouse model

For tumor growth assay, 6 × 10^5^ CFPAC-1 cells with sh-circRTN4 or scramble control (shSCR) were resuspended in 1× PBS with 20% matrigel (Corning, New York, NY, USA) and were injected subcutaneously into the right flank of the randomized male BALB/c nude mice aged 4 to 6 weeks (seven mice per group) [9]. After tumor formation, tumor growth was monitored every 3–4 days, and the tumor volume was measured and calculated by the equation: volume = (Length x width^2^) / 2. Finally, mice were sacrificed, and tumors were excised. Tumor weight was measured. Tumor tissues were collected for analysis.

For tumor metastasis assay, 5 × 10^5^ CFPAC-1 cells with sh-circRTN4 or shSCR were resuspended in 1× PBS with 20% matrigel and were injected orthotopically to the head of the pancreas of randomized male BALB/c nude mice aged 4 to 6 weeks (four mice per group) [9]. The establishment and growth of tumor were monitored every 3–4 days. When the experiment reached the endpoint, mice were sacrificed. Tumors and organs were collected and examined for metastasis.

### Protein stability assay

Analysis of protein stability was performed using cycloheximide (CHX) (Sigma) [13]. After circRTN4 knockdown in PANC-1 cells for 72 h, cells were treated with 100 μg/ml CHX for 2, 4 and 8 h. The level of RAB11FIP1 and GAPDH at each time-point were analyzed by immunoblotting.

### Immunoblotting

The whole cell extract was prepared by lysing cells in RIPA lysis buffer with proteinase inhibitors (Roche, Basel, Switzerland) and phosphatase inhibitor (Thermo Fisher Scientific, Waltham, MA, USA). Proteins were resolved by SDS-PAGE at different percentages, transferred to PVDF membrane and immunoblotted overnight at 4 °C with antibodies against HOXA13 (rabbit; ab106503 Abcam, Cambridge, UK; 1:1000); RAB11FIP1 (rabbit; 16,778–1-AP Proteintech, Rosemont, IL, USA; 1:1000); N-cadherin (mouse; 14–3259 eBioscience, Waltham, MA, USA; 1:1000); PARP (rabbit; #9542 Cell Signaling Technology, Danvers, MA, USA; 1:1000); Bcl-2 (rabbit; 04–436 Millipore, Burlington, MA, USA; 1:1000); and GAPDH (rabbit; #5174 Cell Signaling Technology; 1:1000). Chemiluminescent signals were developed using Clarity™ Western ECL Substrate (Bio-Rad, Hercules, CA, USA).

### Immunohistochemical staining

Immunohistochemical staining was performed using Histostain-Plus IHC Kit, HRP, broad spectrum (Life Technologies, Carlsbad, CA, USA) [14]. The sections were probed with antibodies against Ki67 (rabbit; NB500–170 Novus Biologicals, Centennial, CO, USA; 1:100); Bcl-2 (rabbit; 04–436 millipore; 1:100); HOXA13 (rabbit; ab106503 Abcam; 1:100); N-cadherin (mouse; 14–3259 eBioscience; 1:100), were counter-stained with hematoxylin, and were mounted. A scoring system, based on the percentage of positive cells and staining intensity under the microscope with 100X magnification, was used to quantify the staining. 4 categories (0, 1, 2, and 3) were demoted as 0%, 1–10%, 10–50, and > 50% respectively.

### Identification of circRTN4-interacting proteins

CircRTN4-interacting proteins in PANC-1 cells were identified as described previously [9]. Briefly, *in vitro* transcribed circRTN4 and two negative controls (RTN4 and circGreen Fluorescent Protein (circGFP)) were used incubated with PANC-1 cell lysate at 4 °C overnight with rotation. The pulled-down proteins were identified by Dionex Ultimate3000 nanoRSLC system coupled to Thermo Fisher Orbitrap Fusion Tribid Lumos.

### RNA immunoprecipitation

RNA immunoprecipitation was performed by Magna RIP™ RNA-Binding Protein Immunoprecipitation Kit (Millipore) according to the manufacturer’s protocol. Briefly, cells were incubated magnetic beads labelled with antibody against RAB11FIP1 (rabbit; 16,778–1-AP Proteintech) or TWF1 (mouse; sc-376,539 Santa Cruz Biotechnology, TX, USA) overnight at 4 °C. RNA was purified by phenol:chloroform:isoamylalcohol (Invitrogen). qRT-PCR was used to analyze the enrichment of RNAs with target proteins.

### Modelling of the circRTN4-RAB11FIP1 interaction

The 3-Dimensional structure of circRTN4 and RAB11FIP1 were modelled by the computational methods 3dRNA [15] and I-TASSER [16] respectively. Then, PRIdictor [17] and HDOCK [18] with the default settings were used to model the circRTN4-RAB11FIP1.

### Analysis of publicly available datasets

Publicly available circRTN4 expression datasets in colorectal cancer (CRC) (GSE126095) and laryngeal squamous cell carcinoma (GSE142083) were obtained from Gene Expression Omnibus (GEO) [19, 20]. CircRTN4 expression data in different cancer types were obtained from MiOncoCirc [21].

### Statistical analysis

Statistical analysis was performed by GraphPad Prism 7. Two-tailed student’s t-test, chi square t-test and Pearson’s correlation were used as appropriate. Data were shown in mean ± SD. *P*-value of less than 0.05 was considered as statistically significant.

Full methods were described in Supplementary information.

## Results

### CircRTN4 was upregulated in PDAC

**Fig. 1:**
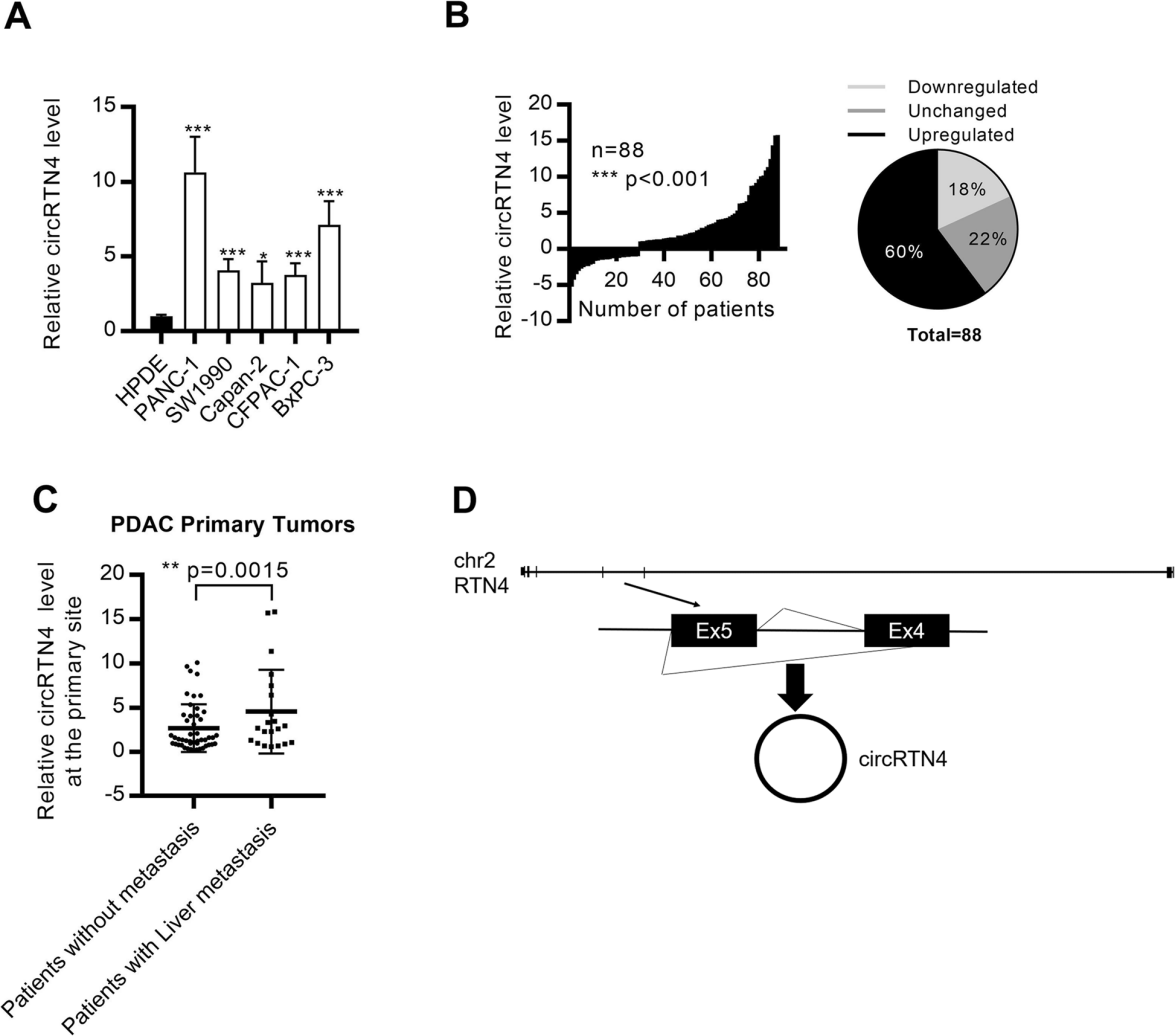
circRTN4 is upregulated in PDAC cells and primary tumors and associates with liver metastasis. **A** CircRTN4 was upregulated in a panel of PDAC cells, compared to non-tumor HPDE cells. **B** CircRTN4 was significantly upregulated in PDAC primary tumors, compared to respective adjacent non-tumor tissues (n = 88). CircRTN4 was upregulated in 60% of the PDAC primary tumors (53 out of 88), while 18% of PDAC patients showed downregulated circRTN4 (16 out of 88), and 22% of PDAC patients showed unchanged circRTN4 (19 out of 88). **C** CircRTN4 was upregulated in PDAC primary tumors from patients with liver metastasis. **D** CircRTN4 was formed by back-splicing of exon 4 and 5 of *RTN4* mRNA. Data represent mean ± SD from at least three independent experiments (*p < 0.05; **p < 0.01***p < 0.001)

CircRNAs profiling in non-tumor HPDE cell and PDAC PANC-1, SW1990 cells was performed to identify circRNAs that play critical roles in PDAC [9]. Among these dysregulated circRNAs, circRTN4 was significantly upregulated in PDAC cells (Fig. 1A). Also, we found that circRTN4 was significantly upregulated in 60% of the PDAC primary tumors (53 out of 88), while 18% of PDAC patients showed downregulated circRTN4 (16 out of 88), and 22% of PDAC patients showed unchanged circRTN4 (19 out of 88) (Fig. 1B). Furthermore, we correlated circRTN4 expression level with clinical characteristics. We found that PDAC patients with liver metastasis had significantly upregulated circRTN4 expression level in their primary tumors (Fig. 1C), suggesting that the upregulated circRTN4 in PDAC primary tumors may involve in liver metastasis. Moreover, male PDAC patients and patients aged 60 or above had further upregulated circRTN4 expression level (Table 1). In addition to PDAC, we found that circRTN4 was frequently upregulated in multiple cancers, including colorectal cancer, laryngeal squamous cell carcinoma, head-neck squamous cell carcinoma, ovarian cancer, and kidney cancer (Supplementary Fig. 1A-C) [19–21]. Collectively, these results suggested that the upregulated circRTN4 play important roles in PDAC.

**Table 1:** Correlations Between circRTN4 and Clinicopathologic Features in PDAC

Clinicopathological Characteristics	Downregulated circRTN4 level	Unchanged circRTN4	Upregulated circRTN4	*P*-value
**Gender**				
Female	12 (34%)	6 (17%)	17 (49%)	**0.0085**
Male	4 (7%)	13 (25%)	36 (68%)	
**Age, years**				
< 60	6 (24%)	9 (36%)	10 (40%)	**0.044**
≥ 60	10 (16%)	10 (16%)	43 (68%)	
**Tumor size, cm**				
≤ 4	6 (12%)	14 (27%)	32 (61%)	0.095
> 4	10 (28%)	5 (14%)	21 (58%)	
**Histological grade**				
Poorly differentiated	2 (50%)	1 (25%)	1 (25%)	0.053
Moderately differentiated	8 (12%)	15 (22%)	44 (66%)	
Well differentiated	4 (40%)	1 (10%)	5 (50%)	
**Tumor stage**				
I-II	13 (18%)	18 (25%)	42 (57%)	0.393
III-IV	3 (21%)	1 (7%)	10 (72%)	

We next characterized circRTN4 as a novel circRNA by examining its physical circular structure. CircRTN4 was formed by the back-splicing of exon 4 and exon 5 of *RTN4* (Fig. 1D). Outward-facing divergent primers and inward-facing convergent primers were designed to examine the circular structure of circRTN4 (Supplementary Fig. 2A). Both the divergent and convergent primers amplified a product of expected size using cDNA from PDAC cells, whereas only the convergent primers amplified a product using genomic DNA from PDAC cells (Supplementary Fig. 2B). The presence of back-splicing junction was further confirmed by Sanger sequencing (Supplementary Fig. 2C). Moreover, we found that circRTN4 was resistant to the digestion by RNase R which specifically degraded linear RNAs but not the circRNAs (Supplementary Fig. 2D). The reduced efficiency of reverse-transcription by oligo-dT primers due to the lack of poly(A) tail also demonstrated the circularity of circRTN4 (Supplementary Fig. 2E). These results confirmed the actual existence of circRTN4 and differentiated it from genomic rearrangement. In addition, the circular structure provided enhanced stability to circRTN4 compared to its parental *RTN4* mRNA. (Supplementary Fig. 2F). Also, coding potential analysis suggested that circRTN4 was lack of protein coding ability (Supplementary Table 3). Furthermore, cellular distribution of circRTN4 was examined by measuring its expression in different cellular compartments. We found that the majority of circRTN4 was present in the cytoplasm of PDAC cells (Supplementary Fig. 2G). Collectively, we characterized the upregulated circRTN4 as a novel non-coding circRNA in PDAC.

### CircRTN4 promoted PDAC cell growth, migration and invasion *in vitro*

**Fig. 2:**
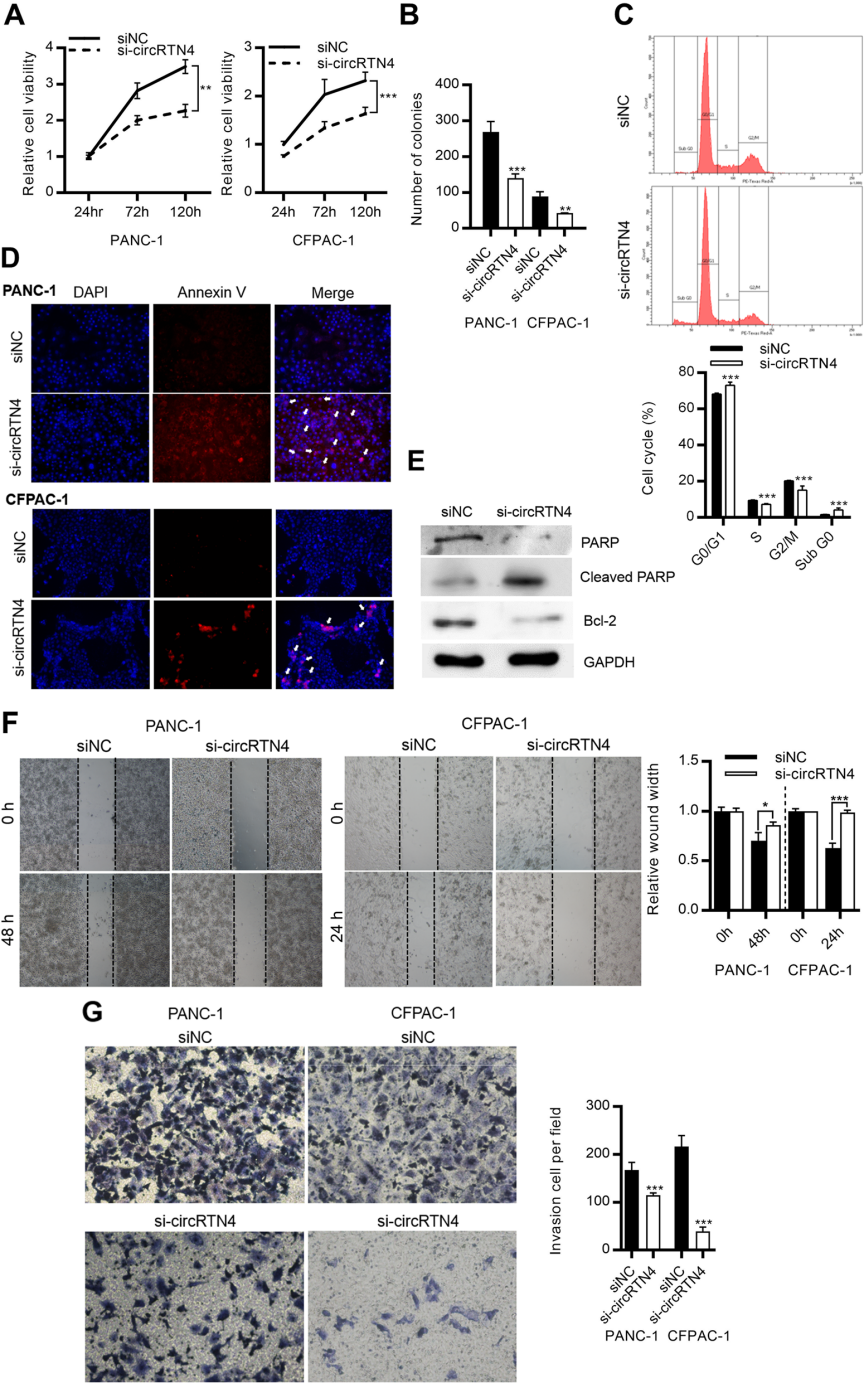
circRTN4 promotes PDAC cell growth, migration, and invasion. **A** CircRTN4 knockdown inhibited PDAC cell growth. **B** CircRTN4 knockdown inhibited PDAC clonogenic ability. **C** Cell cycle was arrested at G0-G1 phase after circRTN4 knockdown in PANC-1 cells. **D** CircRTN4 knockdown resulted in the increase in Annexin V staining (red) in PDAC cells. Nuclei were stained by DAPI (blue). **E** Apoptosis markers PARP was cleaved, and Bcl-2 was reduced after circRTN4 knockdown in PANC-1 cells. **F, G** CircRTN4 knockdown inhibited (**F**) cell migration and (**G**) cell invasion in PDAC cells. Cells in invasion assay were stained by crystal violet. Data represent mean ± SD from at least three independent experiments (*p < 0.05; **p < 0.01; ***p < 0.001)

To examine the functional roles of circRTN4 in PDAC, we used small interfering RNA (siRNA) which specifically targeted the back-splicing junction of circRTN4 without altering the expression of its parental *RTN4* mRNA (Supplementary Fig. 3A and B). CircRTN4 knockdown significantly inhibited PDAC cell growth and clonogenic ability (Fig. 2A and B). The suppression in cell growth was due to cell cycle arrest in G0-G1 phase and the induction of apoptosis (Fig. 2C-E). PDAC cell migration and invasion were also restrained after circRTN4 knockdown (Fig. 2F and G). In addition, circRTN4 knockdown by the lentiviral sh-circRTN4 vector inhibited cell growth, migration, and invasion in PDAC cells (Supplementary Fig. 3C-G). On the other hand, circRTN4 overexpression in non-tumor HPDE cells significantly promoted cell growth, clonogenic ability and cell invasion (Supplementary Fig. 4A-D). Collectively, our results demonstrated the importance of circRTN4 in promoting PDAC cell growth and invasion.

### CircRTN4 promoted PDAC tumor growth and liver metastasis *in vivo*

**Fig. 3:**
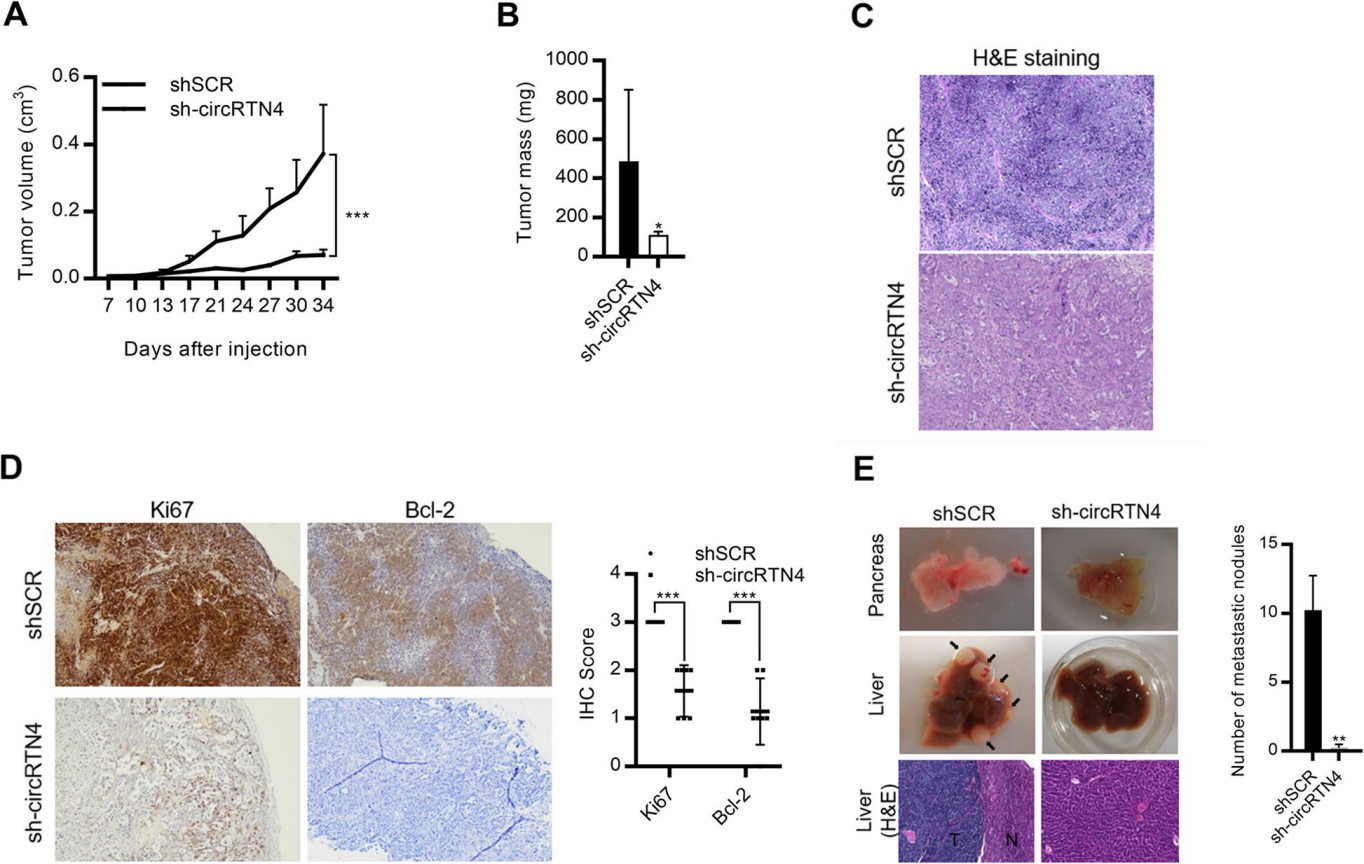
circRTN4 promotes PDAC tumor growth and liver metastasis. **A-C** CircRTN4 knockdown in CFPAC-1 cells inhibited tumor growth. (**A**) tumor volume and (**B**) mass of mice subcutaneous xenograft after knockdown of circRTN4 at day 34. (**C**) Hematoxylin and eosin staining of the subcutaneous xenograft tumors. **D** CircRTN4 knockdown in mice subcutaneous tumors decreased the cell proliferation marker Ki67 and anti-apoptotic protein Bcl-2 level, revealed by immunohistochemical staining. **E** CircRTN4 knockdown in mice orthotopic tumors inhibited liver metastasis. Representative images of liver metastasis were shown. Data represent mean ± SD from at least three independent experiments (*p < 0.05; **p < 0.01; ***p < 0.001)

To investigate the functional roles of circRTN4 in PDAC *in vivo*, mice xenograft model was generated by subcutaneous injection of CFPAC-1 sh-circRTN4 cells. CircRTN4 knockdown by shRNA significantly suppressed tumor growth (Fig. 3A-C). The decrease in cell proliferation marker Ki67 and anti-apoptotic protein Bcl-2 also indicated the inhibition of tumor growth by circRTN4 knockdown (Fig. 3D). Since *in vitro* studies revealed that circRTN4 promoted PDAC migration and invasion, we constructed a metastatic mice model through orthotopic injection of CFPAC-1 sh-circRTN4 cells to the pancreas. We found that circRTN4 knockdown inhibited PDAC metastasis to the liver (Fig. 3E). Collectively, our results suggested the functional importance of circRTN4 in promoting PDAC tumor growth and liver metastasis.

### CircRTN4 promoted lncRNA HOTTIP expression by sponging miR-497-5p

**Fig. 4:**
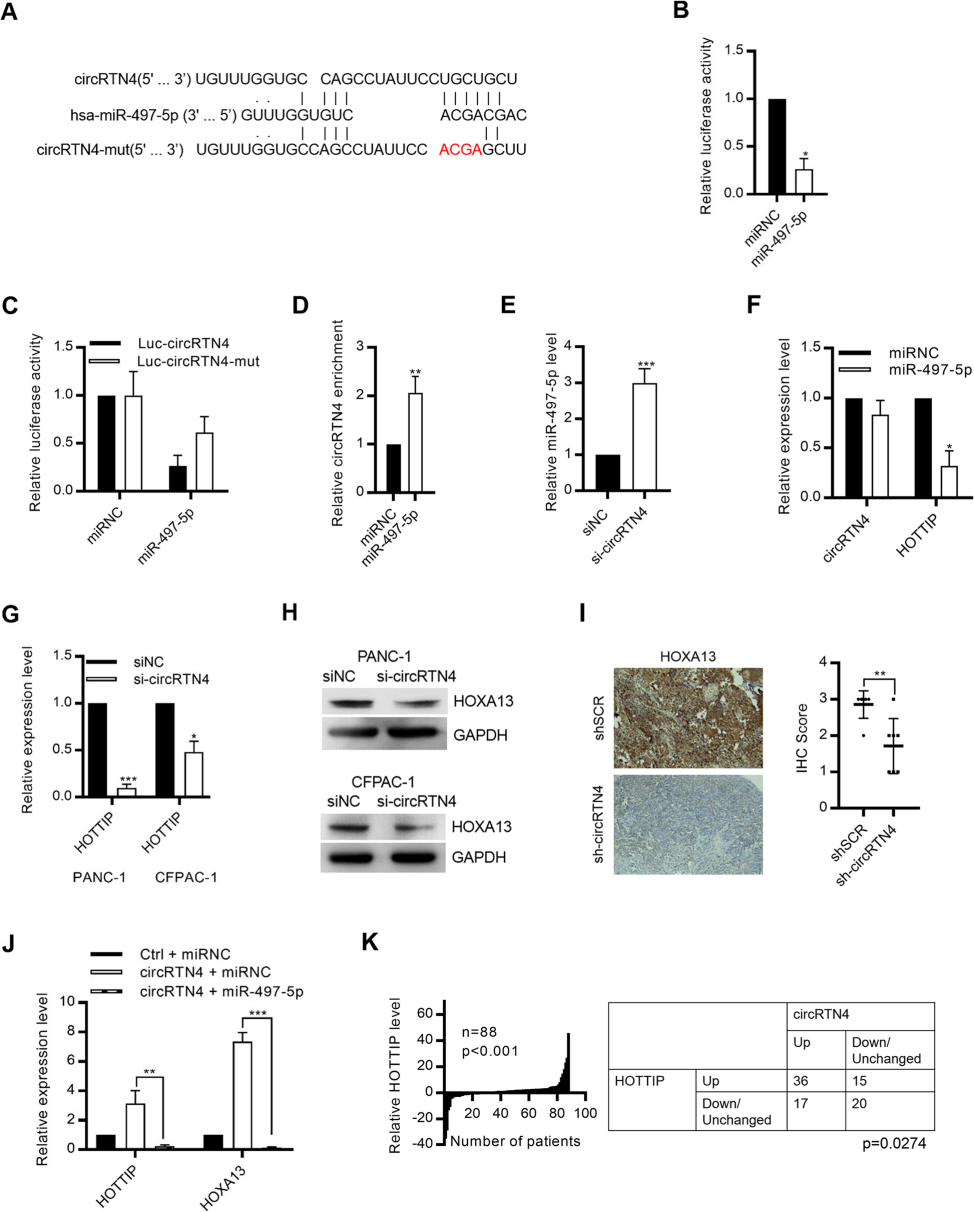
circRTN4 promotes HOTTIP expression by sponging miR-497-5p in PDAC. **A** Schematic diagram showing the putative binding sites of circRTN4 on miR-497-5p. **B** Reduction of luciferase activity of circRTN4-reporter in HEK293 cells co-transfected with miR-497-5p mimics with potential binding sites on circRTN4. **C** Mutating the miR-497-5p binding site on circRTN4-reporter restored the luciferase activity in HEK293 cells co-transfected with miR-497-5p mimics. **D** CircRTN4 was enriched by biotin-labelled miR-497-5p mimics in PANC-1 cells. **E** CircRTN4 knockdown increased the expression of miR-497-5p in PANC-1 cells. **F** Transfecting miR-497-5p mimics did not alter the expression of circRTN4 in PANC-1 cells. **G-H** (**G**) HOTTIP and (**H**) HOAX13 level were reduced after circRTN4 knockdown in PDAC cells. **I** CircRTN4 knockdown in mice subcutaneous tumors inhibited HOXA13 expression, revealed by immunohistochemical staining. **J** HOTTIP and HOXA13 expression were rescued in circRTN4-overexpressing HPDE cells after transfecting miR-497-5p mimics. **K** The upregulated HOTTIP were positively correlated with circRTN4 level in PDAC primary tumors. Data represent mean ± SD from at least three independent experiments (*p < 0.05; **p < 0.01; ***p < 0.001)

We next investigated the detailed mechanism of circRTN4-mediated PDAC progression. CircRNAs frequently function as miRNA sponges in regulating gene expression, we investigated whether circRTN4 also functioned as a miRNA sponge in promoting PDAC progression. Bioinformatics analysis by TargetScan revealed the potential miRNA binding sites on circRTN4 (Fig. 4A) [22]. To prove the circRTN4-miRNA interaction, luciferase assay using pmiR-circRTN4 reporter was performed after transfecting the miRNA mimics. Transfection of miR-497-5p mimics resulted in a reduction in luciferase activity (Fig. 4B). Conversely, mutating the miR-497-5p binding site of the pmiR-circRTN4 reporter could restore the luciferase activity (Fig. 4C). CircRTN4-miR-497-5p interaction was further validated by miRNA pull-down. Biotin-labelled miR-497-5p remarkably enriched circRTN4 in PANC-1 cells (Fig. 4D). These results suggested that circRTN4 interacted with miR-497-5p in PDAC cells. We next studied the functional roles of circRTN4-miR-497-5p interaction in PDAC. We demonstrated that circRTN4 knockdown increased the expression of miR-497-5p, whereas transfecting miR-497-5p mimics did not alter the expression of circRTN4 (Fig. 4E and F). Collectively, our results suggested that circRTN4 functioned as a sponge of miR-497-5p in PDAC cells.

MiR-497-5p functions as a tumor suppressor in PDAC through targeting the oncogenic HOTTIP-HOXA13 pathway [23, 24]. Since circRTN4 acts as a sponge for miR-497-5p, we hypothesized that circRTN4 regulates HOTTIP-HOXA13 pathway via sponging miR-497-5p in PDAC. CircRTN4 knockdown significantly inhibited the expression of HOTTIP and HOXA13 (Fig. 4G-I, Supplementary Fig. 5A). Moreover, circRTN4 overexpression promoted the expression of HOTTIP and HOXA13 in HPDE cells (Supplementary Fig. 5B). In addition, transfection of miR-497-5p mimics rescued the effects of circRTN4 on the expression of HOTTIP and HOXA13 (Fig. 4J). Importantly, circRTN4 level was positively correlated to the upregulated HOTTIP level in PDAC primary tumors (Fig. 4K). These results suggested that circRTN4 functioned as a sponge of miR-497-5p to promote the expression of HOTTIP in PDAC.

### CircRTN4 stabilized RAB11FIP1 to promote EMT

**Fig. 5:**
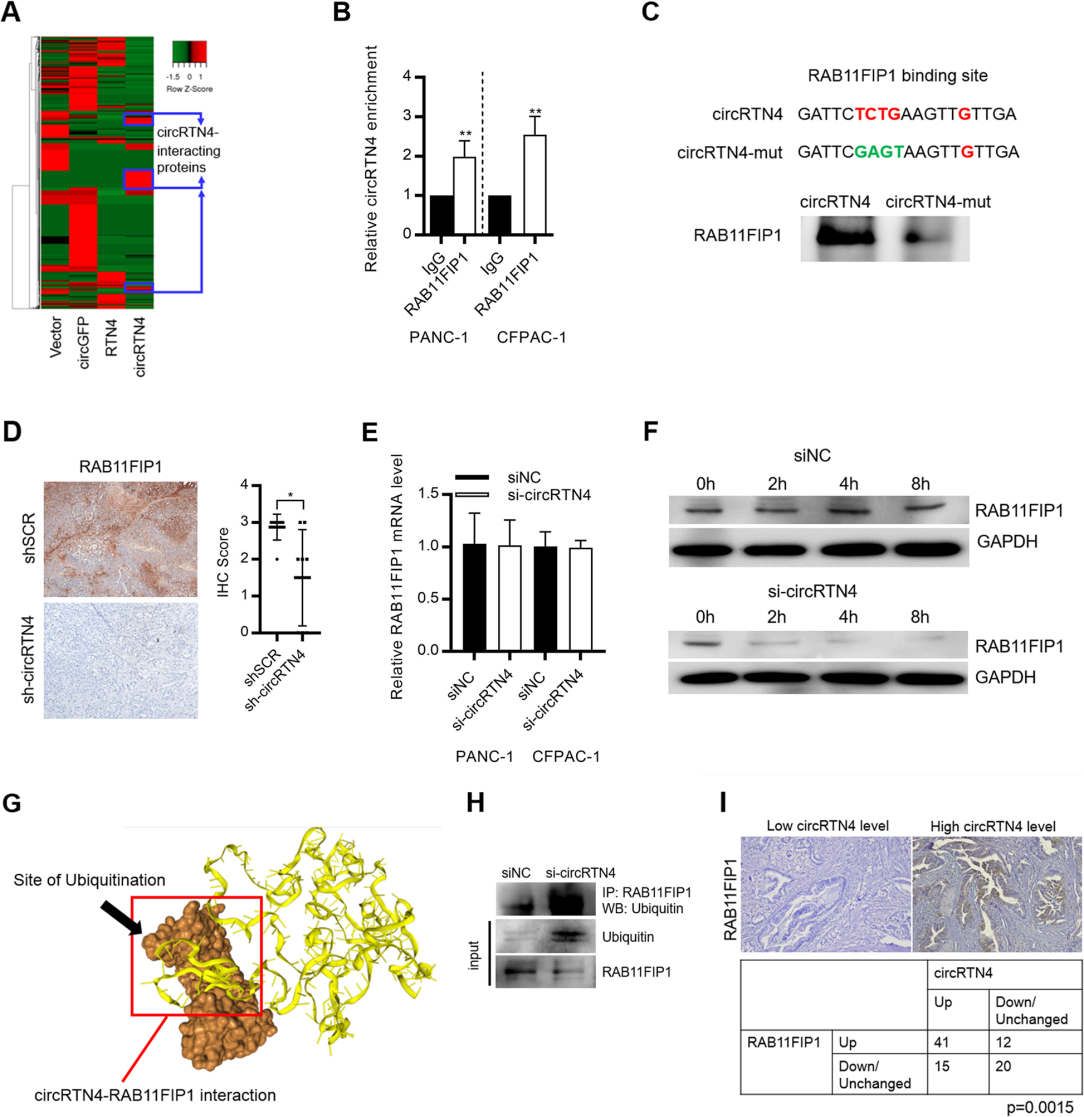
circRTN4 stabilizes RAB11FIP1 by preventing its ubiquitination and degradation. **A** Heat-map showing 99 circRTN4-interacting proteins in PDAC. Biotin-labelled circRTN4, *RTN4* mRNA, circGFP were used to pull down circRTN4-interacting proteins. Mass spectrometry analysis was performed to identify the interacting proteins. **B** CircRTN4 interacted with RAB11FIP1 in PDAC cells, as revealed by RIP assay. **C** Bioinformatics analysis by PRIdictor revealed the RAB11FIP1-binding site (The seed region of interaction was in red) on circRTN4. Mutating RAB11FIP1-binding site on circRTN4 (The mutated seed region of interaction was in green) inhibited circRTN4-RAB11FIP1 interaction in PANC-1 cells, as revealed by circRNA pulldown assay. **D** CircRTN4 knockdown inhibited RAB11FIP1 expression in mice xenograft. **E** CircRTN4 knockdown did not affect RAB11FIP1 mRNA level in PDAC cells. **F** CircRTN4 knockdown decreased the stability of RAB11FIP1 after inhibition of protein synthesis by cycloheximide in PANC-1 cells. **G** 3-Dimensional structure of the circRTN4-RAB11FIP1 interaction revealed that circRTN4 blocked the ubiquitination site Lys578 of RAB11FIP1. **H** Immunoprecipitation with anti-RAB11FIP1 antibody in PANC-1 cells after circRTN4 knockdown, followed by immunoblotting analysis with anti-ubiquitin or anti-RAB11FIP1 antibody. CircRTN4 knockdown increased ubiquitination of RAB11FIP1. **I** RAB11FIP1 expression were upregulated in PDAC primary tumors and was positively correlated with circRTN4 level. Data represent mean ± SD from at least three independent experiments (*p < 0.05; **p < 0.01; ***p < 0.001)

Although circRNAs-miRNAs interaction is the most reported roles of circRNAs in PDAC, many circRNAs are found to have much lower abundance than miRNAs. This suggests that circRNAs may have additional mechanisms in PDAC. Therefore, to further investigate the roles of circRTN4 in PDAC progression, we performed circRTN4-pull down to identify circRTN4-interacting proteins in PDAC cells. *In vitro*-transcribed circRTN4 functioned as a probe to pull down circRTN4-interacting proteins in PDAC cells. Mass-spectrometry analysis identified 99 proteins significantly enriched by circRTN4 (Fig. 5A). In addition, analysis using the STRING database [25] revealed 90 protein-protein interactions (PPI) for the circRTN4-interacting proteins (Supplemental Fig. 6A). Gene ontology analysis also suggested the critical roles of circRTN4-interacting proteins in multiple biological processes, including cadherin binding, cell adhesion and translation initiation (Supplementary Fig. 6B).

Particularly, we found that circRTN4 interacted with RAB11FIP1, which plays important roles in promoting cell migration and invasion in cancers [26–29]. CircRTN4-RAB11FIP1 interaction was further validated by RNA immunoprecipitation assay in PDAC cells (Fig. 5B). Bioinformatics analysis by PRIdictor also revealed the RAB11FIP1-binding site on circRTN4 (Supplementary Fig. 7) [17]. Mutating the RAB11FIP1-binding site on circRTN4 significantly inhibited the circRTN4-RAB11FIP1 interaction in PANC-1 cells (Fig. 5C). These confirmed that circRTN4 interacted with RAB11FIP1 in PDAC cells. We then investigated the functions of this interaction in PDAC. We found that knockdown of circRTN4 downregulated the RAB11FIP1 expression, without affecting its mRNA level (Fig. 5D and E). Importantly, circRTN4 knockdown decreased the stability of RAB11FIP1 in PDAC cells (Fig. 5F). We then further investigated the detailed mechanism on how circRTN4 regulated the stability of RAB11FIP1. Previous study showed that ubiquitination at the Lysine578 (Lys578) residue of RAB11FIP1 is required for its degradation [30]. Notably, we revealed the 3-Dimensional structure of circRTN4-RAB11FIP1 interaction by HDOCK [18], and found that circRTN4 blocked the ubiquitination site Lys578 on RAB11FIP1 (Fig. 5G). Also, circRTN4 knockdown in PDAC cells promoted the ubiquitination of RAB11FIP1 (Fig. 5H). Importantly, circRTN4 level was positively correlated to the RAB11FIP1 level in PDAC primary tumors (Fig. 5I). These suggested that circRTN4 interacted with RAB11FIP1 and enhanced the stability of RAB11FIP1 through inhibiting its ubiquitination in PDAC.

**Fig. 6:**
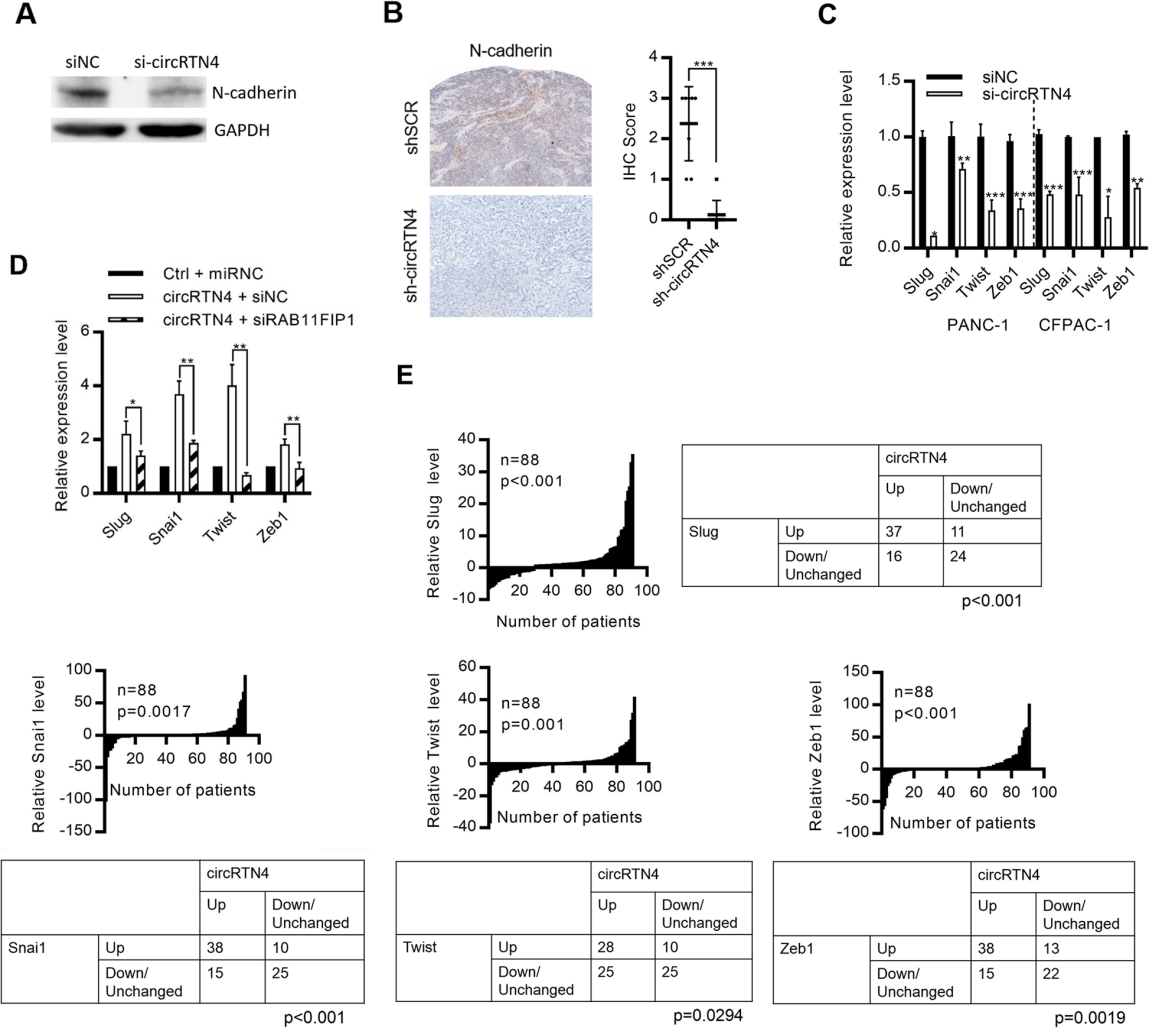
circRTN4 stabilizes RAB11FIP1 to promote EMT in PDAC. **A** N-cadherin expression was inhibited after circRTN4 knockdown in PANC-1 cells. **B** CircRTN4 knockdown in mice subcutaneous tumors inhibited N-cadherin expression. **C** Expression of EMT markers Slug, Snai1, Twist and Zeb1 were reduced after circRTN4 knockdown in PDAC cells. **D** Expression of Slug, Snai1, Twist and Zeb1 in circRTN4-overexpressing HPDE cells were rescued after RAB11FIP1 knockdown. **E** Slug, Snai1, Twist and Zeb1 expression were upregulated in PDAC primary tumors and were positively correlated with circRTN4 level. Data represent mean ± SD from at least three independent experiments (*p < 0.05; **p < 0.01; ***p < 0.001)

RAB11FIP1 plays important roles in promoting cancer migration and invasion through regulating the expression of EMT-related proteins (Supplementary Fig. 8A -C) [31, 32]. Therefore, we hypothesized that circRTN4 stabilizes RAB11FIP1 in regulating EMT in PDAC. We demonstrated that circRTN4 knockdown inhibited the expression of N-cadherin in PDAC cells and mice tumors (Fig. 6A and B). Also, we found the differential expression of EMT-related transcription factors in circRTN4-depleted PDAC cells. CircRTN4 knockdown inhibited expression of transcription factors Snail Family Transcriptional Repressor 2 (Slug), Snail Family Transcriptional Repressor 1 (Snai1), Twist Family BHLH Transcription Factor 1 (Twist) and Zinc Finger E-Box Binding Homeobox 1 (Zeb1) (Fig. 6C). In addition, knockdown of RAB11FIP1 rescued the effects of circRTN4 on the expression of Slug, Snai1, Twist and Zeb1 (Fig. 6D). Accordingly, we demonstrated that Slug, Snai1, Twist and Zeb1 were upregulated in PDAC primary tumors (Fig. 6E). A positive correlation was also found between Slug, Snai1, Twist, Zeb1 and circRTN4 expression (Fig. 6E). Taken together, our results suggested that circRTN4 stabilized RAB11FIP1 by blocking its ubiquitination to promote the expression of N-cadherin, Slug, Snai1, Twist and Zeb1 for EMT in PDAC.

## Discussion

CircRNAs, as the novel member of non-coding RNA family, play vital roles in multiple cancers [6–8, 33]. With limited knowledge on the roles of circRNAs in PDAC, we previously performed circRNA sequencing to identify differentially expressed circRNAs in PDAC cells [9]. One of the upregulated circRNAs, circRTN4, was examined in detail in the current study. RTN4 (also named as Nogo), which is a myelin-associated endoplasmic reticulum protein, is well-known for its function in the nervous system as an inhibitor of axon regeneration [34–36]. Moreover, differential expression of RTN4 is found in multiple cancers, which may function as a potential prognostic marker for gastric cancer [37–40]. Also, RTN4 promotes cancer progression by facilitating tumor proliferation and drug resistance [38, 39]. Notably, the exon 2 and 3 of *RTN4* mRNA can be circularized to form a protein-coding circRNA: hsa_circ_0054598 in brain [41, 42]. Also, exosomal hsa_circ_0054598 may be used to treat osteoporosis [43]. In the current study, we demonstrated that another circRTN4, which is formed by the back-splicing of exon 4 and exon 5 of *RTN4* mRNA, is significantly upregulated in PDAC cells and primary tumors. Also, PDAC patients with liver metastasis have high circRTN4 level in their primary tumors. Furthermore, the biological roles of circRTN4 in promoting PDAC cell growth and liver metastasis are explored in knockdown and overexpression experiments.

CircRNAs can reportedly interact with and sponge miRNAs in regulating gene expression in PDAC. circRNA_100782 promotes PDAC tumor growth by sponging tumor suppressor miR-124 [44]. hsa_circRNA_0007334 inhibits miR-144-3p and miR-577 to upregulate collagen type I alpha 1 chain and matrix metallopeptidase 7 [45]. ciRS-7 and circPDE8A promote PDAC cell growth and invasion by targeting miR-7 and miR-338 respectively [46, 47]. CircRHOT1 promotes PDAC cell invasion by sponging miR-26b, miR-125a, miR-330 and miR-382 [48]. Consistently, we demonstrated that circRTN4 sponged tumor-suppressive miR-497-5p in PDAC. Notably, we previously reported that miR-497-5p inhibited PDAC progression through suppressing the oncogenic HOTTIP-HOXA13 pathway [24]. In current study, we demonstrated that circRTN4 is the regulator of HOTTIP, which is one of the well-characterized lncRNA. HOTTIP complexed with MLL1 and WDR5 to promote gene-activating H3K4 trimethylation at the gene promoter [49, 50]. In many cancer types, HOTTIP is frequently upregulated and plays critical roles in promoting cancer progression [51–54]. Upregulation of HOTTIP promotes cancer growth and invasion in colorectal cancer [51]. Also, HOTTIP promotes tumor growth and metastasis through regulating HOXA genes in hepatocellular carcinoma [54]. We demonstrated that the upregulated HOTTIP promoted PDAC cell growth and invasion under the negative regulation by miR-497-5p [24]. In current study, we identified a novel circRTN4-miR-497-5p-HOTTIP pathway in PDAC. We demonstrated that circRTN4 promotes HOTTIP expression through inhibiting miR-497-5p. This demonstrated a novel role of circRNA in regulating the expression of lncRNA through circRNA-miRNA-lncRNA pathway in cancer.

Functioning as miRNAs sponges is the first identified role of circRNAs [55]. Many studies have also demonstrated the importance of miRNAs sponges in cancer development. Notably, there is growing evidence to show that circRNAs regulate gene expression through circRNA-protein interactions, suggesting circRNAs may have more than one mode of action. circADD3 complexes with CDK1 to protect EZH2 from degradation [31]. circCTNNB1 interacts with DDX3 and YY1 to promote gene expression [56]. circFOXK3 binds and inhibits the activities of CDK2 and p21 [57]. We also demonstrated that circFOXK2 complexes with YBX1 and hnRNPK in promoting the expression of oncogenic proteins NUF2 and PDXK in PDAC [9]. Herein, circRNA pull-down and mass spectrometry were performed to identify circRTN4 binding proteins. We identified 99 circRTN4-interacting proteins that are involved in several biological processes, including cell adhesion and cadherin binding. We demonstrated that circRTN4 interacts with RAB11FIP1 in PDAC cells. Most importantly, our data showed that circRTN4 stabilizes RAB11FIP1 by preventing its ubiquitination. RAB11FIP, as a member of GTP-bound Rab11 effectors, regulated cell polarity through participating in the vesicle trafficking system [58–60]. Several studies reported that the upregulation of RAB11FIP1 and RAB11FIP2 and their interacting partner RAB11a promotes cancer migration and invasion [26–29]. RAB11FIP1 protects integrins from degradation, regulates cadherin recycling and promotes the expression of EMT-related transcription factors [27, 29, 31, 32, 61, 62]. In this study, we found that circRTN4 promotes EMT via RAB11FIP1-mediated upregulation of N-cadherin, Slug, Snai1, Twist and Zeb1 in PDAC. These may suggest the importance of circRTN4 in promoting cancer metastasis by EMT in PDAC.

## Conclusion

In summary, our study emphasizes the significance of circRTN4 in PDAC progression. CircRTN4 is ectopically expressed in many cancers, including PDAC. Overexpression and knockdown studies indicated the upregulated circRTN4 promotes PDAC cell growth and liver metastasis. Mechanistically, circRTN4 promotes expression of oncogenic HOTTIP by sponging miR-497-5p. This reveals a novel circRNA-miRNA-lncRNA pathway in promoting PDAC progression. Furthermore, we provide a new perspective on the tumorigenic ability of circRTN4 through stabilizing RAB11FIP1 by blocking its ubiquitination to enhance the expression of EMT markers in PDAC. This highlights the potential of circRTN4 as a novel biomarker and a potential therapeutic target for PDAC.

## Supplementary Information

**Additional file 1: Supplementary methods**, **Supp Figure 1.** circRTN4 is upregulated in cancer. **Supp Figure 2.** Characterization of circRTN4 in PDAC cells. **Supp Figure 3.** circRTN4 promotes PDAC cell growth, migration and invasion. **Supp Figure 4.** circRTN4 promotes HPDE cell growth and invasion. **Supp Figure 5.** circRTN4 promotes the expression of oncogenic HOTTIP-HOXA13 pathway in PDAC. **Supp Figure 6.** Identification of circRTN4-interacting proteins in PDAC. **Supp Figure 7.** Prediction of circRTN4-RAB11FIP1 interaction. **Supp Figure 8.** RAB11FIP1 promotes the expressions of N-cadherin and EMT-related transcription factors in PDAC. **Supplementary Table 1.** siRNA sequences for gene knockdown and miRNA mimic sequences. **Supplementary Table 2.** Oligos used in this study. **Supplementary Table 3.** Coding potential analysis of circRTN4 by Coding Potential Assessment Tool.

## Data Availability

CircRNA sequencing data are available in the NCBI Gene Expression Omnibus under accession number GSE135731. The GEO accession token for reviewer was gdenssignnolzyx. Mass spectrometry data of circRNA pull-down are available via ProteomeXchange with identifier PXD015048. The ProteomeXchange reviewer username and password were reviewer30182@ebi.ac.uk; 4RHLjCxH.

## Abbreviations

circRNA: Circular RNA
Ctrl: Control
DMEM: Dulbecco’s modified Eagle’s medium
EMT: Epithelial-Mesenchymal Transition
FBS: Fetal bovine serum
FFPE: Formalin-fixed, paraffin-embedded
GAPDH: Glyceraldehyde-3-Phosphate Dehydrogenase
HOTTIP: HOXA Distal Transcript Antisense RNA
HOXA13: Homeobox A13
lncRNA: Long non-coding RNA
miRNA: Micro RNA
MTT: 3-(4, 5-Dimethylthiazol-2-yl)-2, 5-diphenyltetrazolium bromide
NC: Negative control
PARP: Poly(ADP-Ribose) Polymerase 1
PBS: Phosphate buffered saline
PCR: Polymerase chain reaction
PDAC: Pancreatic ductal adenocarcinoma
PPI: Protein-protein interaction
qRT-PCR: Quantitative reverse transcription PCR
RIP: RNA immunoprecipitation
RAB11FIP1: RAB11 Family Interacting Protein 1
RTN4: Reticulon 4
shRNA: Small hairpin RNA
siRNA: Small interfering RNA
Slug: Snail Family Transcriptional Repressor 2
Snai1: Snail Family Transcriptional Repressor 1
Twist: Twist Family BHLH Transcription Factor 1
Zeb1: Zinc Finger E-Box Binding Homeobox 1
