# Supplementary material for: CircRTN4 promotes pancreatic cancer progression through a novel CircRNA-miRNA-lncRNA pathway and stabilizing epithelial-mesenchymal transition protein
Source: Mol Cancer. 2022 Jan 4;21:10. doi: 10.1186/s12943-021-01481-w (PMC8725379; doi:10.1186/s12943-021-01481-w)
Supplement: Supplementary file 1 — Additional file 1: Supplementary methods, Supp Figure 1. circRTN4 is upregulated in cancer. Supp Figure 2. Characterization of circRTN4 in PDAC cells. Supp Figure 3. circRTN4 promotes PDAC cell growth, migration and invasion. Supp Figure 4. circRTN4 promotes HPDE cell growth and invasion. Supp Figure 5. circRTN4 promotes the expression of oncogenic HOTTIP-HOXA13 pathway in PDAC. Supp Figure 6. Identification of circRTN4-interacting proteins in PDAC. Supp Figure 7. Prediction of circRTN4-RAB11FIP1 interaction. Supp Figure 8. RAB11FIP1 promotes the expressions of N-cadherin and EMT-related transcription factors in PDAC. Supplementary Table 1. siRNA sequences for gene knockdown and miRNA mimic sequences. Supplementary Table 2. Oligos used in this study. Supplementary Table 3. Coding potential analysis of circRTN4 by Coding Potential Assessment Tool. [file 12943_2021_1481_MOESM1_ESM.docx]

**Supplementary methods**

**Characterization of circRTN4 in PDAC**

The circular structure of circRTN4 was confirmed as described previously.^9^ In brief, for RNase R digestion assay, RNA was digested with or without RNase R (Lucigen) at 37 °C for 15 mins. For circRTN4 stability assay, transcription was blocked by 2 μg/mL actinomycin D (Sigma) for 4 h, 12 h and 24 h. For circRTN4 localization study, nuclear fraction and cytoplasmic fraction of PANC-1 cells were isolated by NE-PER Nuclear and Cytoplasmic Extraction Reagents (Thermo Fisher Scientific). RNA was then purified by TRIZOL Reagent.

**Quantitative reverse transcription PCR (qRT-PCR)**

Reverse transcription of RNA was performed by High-Capacity cDNA Reverse Transcription Kit (Applied Biosystems, Waltham, MA, USA). Reverse transcription of miRNA was performed by Mir-X™ miRNA First-Strand Synthesis Kit (Takara, Japan). qRT-PCR was performed by ABI 7900HT Real-Time PCR system using SYBR Green PCR Master Mix (Applied Biosystems). The primers used in this study were listed in Supplementary Table 2.

**Cell viability assay**

3-(4,5-dimethylthiazol-2-yl)-2,5-diphenyltetrazolium bromide (MTT) cell viability assay was performed by seeding 2500 cells in a 96-well plate. After 24, 72 and 120 h, the medium was removed and incubated with 0.65 mg/mL MTT in DMEM. After 2 h, the mixture was removed, and the insoluble formazan was solubilized by adding 120 μL DMSO. After 5 mins, absorbance at 570 nm was measured using microplate spectrophotometer (Biorad).

**Anchorage-dependent colony formation assay**

Anchorage-dependent colony formation assay was performed by seeding 1000 cells in a 6-well plate. After growing the cells for 2 weeks, the cells were fixed with 37% formaldehyde for 5 mins, and stained by 0.5% crystal violet in methanol. After staining for 5 mins, excess stain was removed by rinsing in PBS. Photos were taken and number of colonies was counted.

**Cell migration assay**

Wound healing cell migration assay was performed by seeding 500,000 cell/ mL in a 3-well silicone insert with a defined cell-free gap in a 24-well plate. On the next day, in which cells reached 90% confluency, the insert and medium were removed and culture medium without serum was added. Wound width was measured at 0, 24 and 48 h.

**Cell invasion assay**

Upper chamber of the trans-well insert with pore size of was coat with Matrigel (Corning, Glendale, AZ, USA) and was placed in a 37°C incubator overnight. Then the unsolidified Matrigel was removed and was seeded with 25000 cells in culture medium without serum. Then the insert with seeded cells was placed in a plate well with complete culture medium. After 24 to 72 h, the trans-well was fixed with 3.7 % formaldehyde for 5 mins, permeabilized by methanol for 15 mins, and was then stained by 0.5 % crystal violet for 20 mins. The cells in the upper chamber (non-invasive cells) were removed and the invaded cells were counted.

**Cell cycle analysis**

After knockdown of circRNAs for 72 h, cells were trypsinized and washed twice with 2 % FBS in PBS. Cells were then fixed with 70 % ethanol at 4 °C for 3 h. After ethanol fixation, cells were washed twice with 2 % FBS in PBS, followed by treatment with 50 μg/mL RNase A (Thermo Fisher Scientific) and 10 μg/mL propidium iodide at 37 °C for 15 mins. Cell cycle was analyzed by BD LSR Fortessa Cell Analyzer (BD Biosciences, Franklin Lakes, NJ, USA). The cell cycle phase distribution and proportion of apoptotic cells were analyzed using BD FACSDiva software.

**Apoptosis assay**

Cell apoptosis assay after knockdown of circRNAs was performed by Annexin V-Cy5 Apoptosis Detection Kit (BioVision, Milpitas, CA, USA). Cells in 24-well plate were washed twice with ice-cold PBS. Then cells were stained by DAPI and 5 μL Annexin V in 500 μL 1X Annexin V Binding Buffer. After incubation at room temperature for 5 mins in dark, apoptotic cells were analyzed under fluorescent microscope.

**miRNA Pull down**

miRNA pull down assay was performed as described previously. Briefly, PANC-1 cells were transfected with 3’-end biotinylated miRNA mimics. After 24 h transfection, the cells were lysed, and the biotin-labelled miRNA mimics were isolated by Dynabeads MyOne Streptavidin C1 beads (Thermo Fisher Scientific) at 4°C for 4h with rotation. Biotin-labelled miRNAs and their interacting RNAs were isolated by TRIZOL Reagent. Detection of miRNA interacting RNAs was performed by RT-qPCR.

**Luciferase assay**

HEK293 cells were co-transfected with pmiR-Reporter plasmid, Renilla luciferase reporter plasmid and miRNA mimics. After 72 h, luciferase activity was measured with Nano-Glo® Dual-Luciferase® Reporter Assay 18 System (Promega). For comparison, Firefly luciferase activity was first normalized with Renilla luciferase activity. Then, the effect of each miRNA mimics on pmiR-Reporter with circRTN4 sequence was normalized with pmiR-Reporter without circRTN4. Finally, fold change was calculated by comparing the effect of miRNA mimics to miRNC.


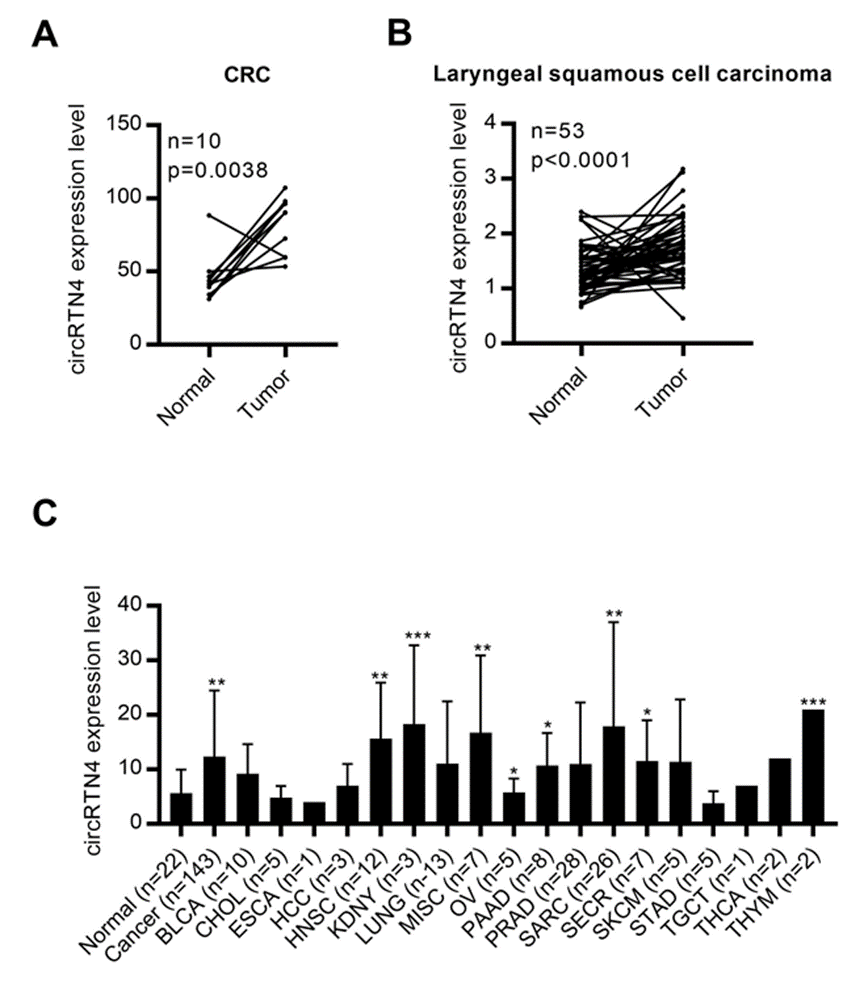


**Supp Figure 1. circRTN4 is upregulated in cancer.** (A-B) CircRTN4 was upregulated in (A) colorectal cancer (CRC) (GSE126095) and (B) laryngeal squamous cell carcinoma (GSE142083). (C) CircRTN4 was upregulated in head-neck squamous cell Carcinoma (HNSC), and kidney cancer (KIDY), ovarian cancer (OV), pancreatic adenocarcinoma (PAAD), sarcoma (SARC), tumors of secretory organs (SECR), thymoma (THYM), compared to normal tissues, from MiOncoCirc dataset. Data represent mean ± SD (*p<0.05; **p<0.01***p<0.001).

**
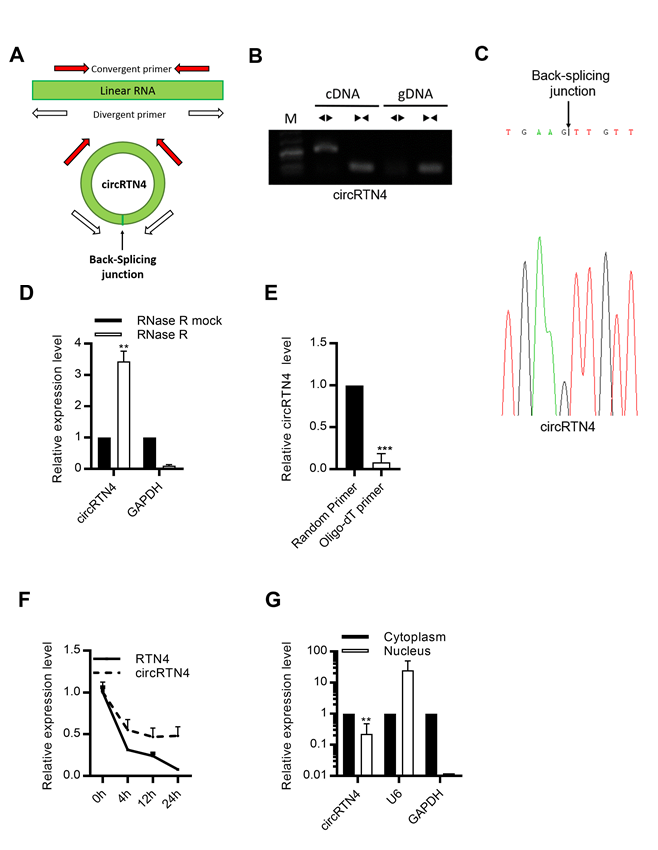
**

**Supp Figure 2. Characterization of circRTN4 in PDAC cells.** (A) Schematic diagram illustrating the design of divergent and convergent primers for amplifying circRTN4. (B) Detection of circRTN4 using divergent and convergent primers with PANC-1 cDNA and gDNA. (C) Validation of the back-splicing junction of circRTN4 by Sanger sequencing. (D) circRTN4 was resistant to the digestion by RNase R. (E Reverse-transcription efficiency of circRTN4 was reduced when oligo-dT primer was used. (F) circRTN4 was relatively more stable than its parental *RTN4* after inhibition of transcription by Actinomycin D. (G) Analysis of cellular location of circRTN4 in PANC-1 by qRT-PCR. circRTN4, U6 and GAPDH levels in nuclear fractions were compared to respective levels in cytoplasmic fraction. Data represent mean ± SD from at least three independent experiments (**p<0.01; ***p<0.001).

**
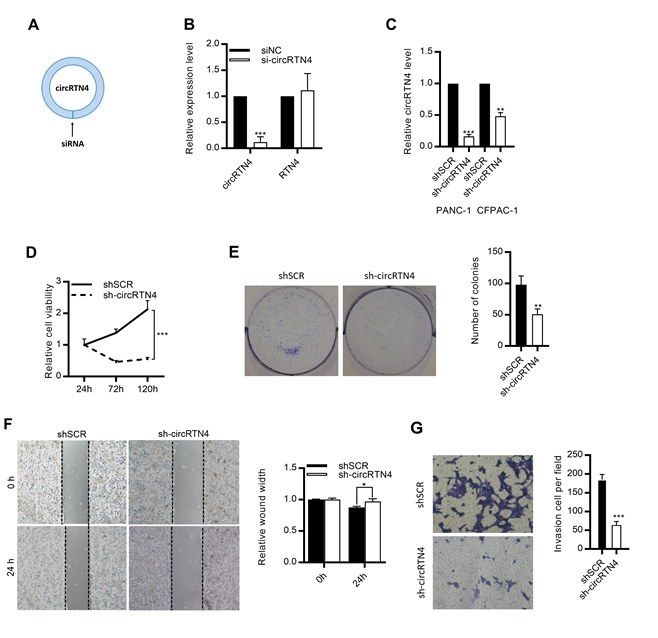
Supp Figure 3. circRTN4 promotes PDAC cell growth, migration and invasion.** (A) Schematic diagram illustrating the design of siRNA which specifically targeted back-splicing junction of circRTN4. (B) Validation of knockdown efficiency and specificity of siRNA on circRTN4 after transfecting PANC-1 cells for 72h. (C) Validation of knockdown efficiency of circRTN4 by stable transfection of shRNA in PDAC cells. (D) CircRTN4 knockdown by shRNA inhibited PANC-1 cell growth. (E) CircRTN4 knockdown by shRNA inhibited clonogenic ability in CFPAC-1 cells. (F-G) Stable circRTN4 knockdown by shRNA inhibited (F) migration and (G) invasion in CFPAC-1 cells. Cells in invasion assay were stained by crystal violet. Data are mean ± SD from at least three independent experiments (*p<0.05; **p<0.01; ***p<0.001)


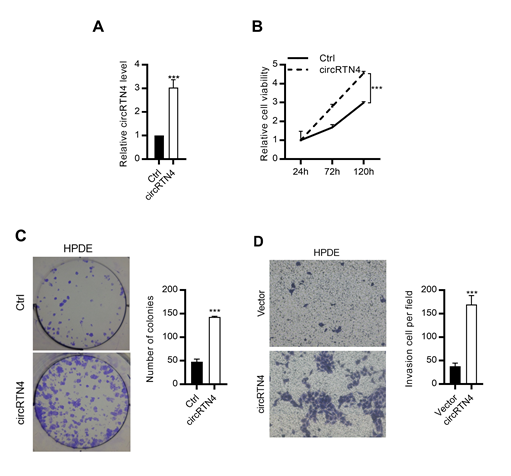


**Supp Figure 4. circRTN4 promotes HPDE cell growth and invasion.** (A) Analysis of circRTN4 expression level after circRTN4 overexpression in HPDE cells. (B) CircRTN4 overexpression promoted cell growth in HPDE cells. (C) CircRTN4 overexpression promoted clonogenic ability in HPDE cells. (D) CircRTN4 overexpression promoted cell invasion in HPDE cells. Cells were stained by crystal violet. Data are mean ± SD from at least three independent experiments (***p<0.001)


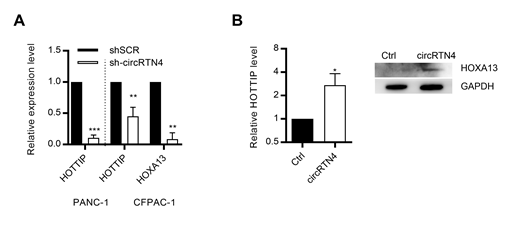


**Supp Figure 5. circRTN4 promotes the expression of oncogenic HOTTIP-HOXA13 pathway in PDAC.** (A) Expression levels of HOTTIP and HOXA13 were decreased after stable circRTN4 knockdown by shRNA in PDAC cells. (B) CircRTN4 overexpression promoted the expression levels of HOTTIP and HOXA13 in HPDE cells. Data are mean ± SD from at least three independent experiments (*p<0.05; **p<0.01; ***p<0.001)


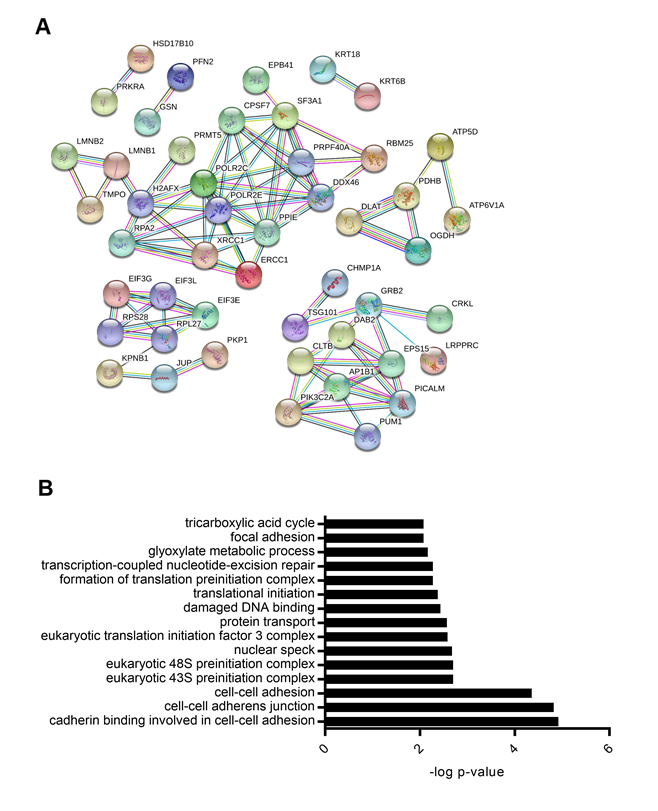


**Supp Figure 6. Identification of circRTN4-interacting proteins in PDAC.** (A) Analysis of protein-protein interactions of circRTN4-interacting proteins by STRING 11. Each node represents a protein, and each edge represents a protein-protein interaction. Color of each edge denotes the type of evidence of the interaction. (Confidence level was set at high confidence of 0.7). (B) Gene Ontology analysis of the circRTN4-interacting proteins in PDAC. The top 15 Gene Ontology clusters were listed.


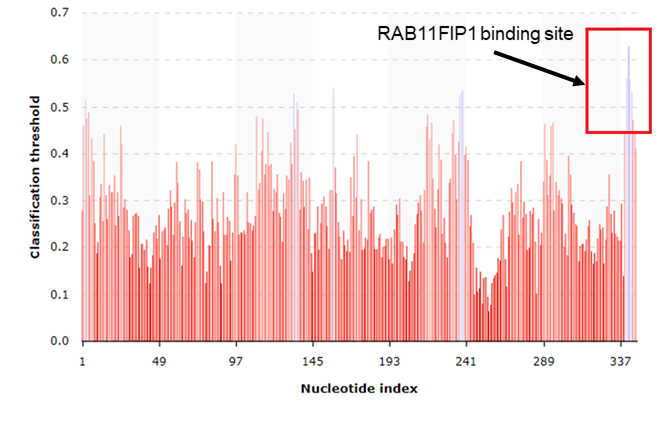


**Supp Figure 7. Prediction of circRTN4-RAB11FIP1 interaction.** The potential RAB11FIP1 binding sites on circRTN4 were predicted by PRIdictor.


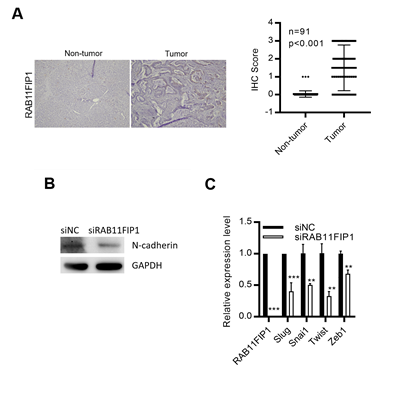


**Supp Figure 8. RAB11FIP1 promotes the expressions of N-cadherin and EMT-related transcription factors in PDAC.** (A) RAB11FIP1 was upregulated in PDAC primary tumors. (B) N-cadherin was downregulated after RAB11FIP1 knockdown in PANC-1 cells. (C) Expressions of Slug, Snai1, Twist and Zeb1 were inhibited after RAB11FIP1 knockdown in PANC-1 cells. Data are from at least three independent experiments mean ± SD (*p<0.05; **p<0.01; ***p<0.001)

**Supplementary Table 1. siRNA sequences for gene knockdown and miRNA mimic sequences**

|  | Sense | Antisense |
| --- | --- | --- |
| si-circRTN4 | GAUUCUCUGAAGUUGUUGATT | UCAACAACUUCAGAGAAUCTT |
| siRAB11FIP1 | CGAUAAGCAAGAAGGAGUUTT | AACUCCUUCUUGCUUAUCGTT |
| hsa-miR-497-5p | CAGCAGCACACUGUGGUUUGU | AAACCACAGUGUGCUGCUGUU |

**Supplementary Table 2. Oligos used in this study**

| circRTN4-F | GCATTGTGAGCGTAACAGCC |
| --- | --- |
| circRTN4-R | AGCTTGGATCACACCCTTGT |
| RTN4-F | GCATTGTGAGCGTAACAGCC |
| RTN4-R | AGCTTGGATCACACCCTTGT |
| HOTTIP-F | ACGCATATTCACGCATCA |
| HOTTIP-R | TTACCAAGCCACAGGAGA |
| HOXA13-F | TGGAACGGCCAAATGTACTG |
| HOXA13-R | TGGCGTATTCCCGTTCAAGT |
| RAB11FIP1-F | GGATGTCTCCGAATCTTCCA |
| RAB11FIP1-R | CCGTCATCAGAGACAGCAAA |
| Slug-F | AGATGCATATTCGGACCCAC |
| Slug-R | CCTCATGTTTGTGCAGGAGA |
| Snai1-F | AGAGCTGACCTCCCTGTCA |
| Snai1-R | TGAAGTAGAGGAGAAGGACGAA |
| Twist-F | GTCCGCAGTCTTACGAGGAG |
| Twist-R | CCAGCTTGAGGGTCTGAATC |
| Zeb1-F | CATCTTGAGCTGAATTTGGGTAACA |
| Zeb1-R | CCTGAAATGACCTGAAGCATGAA |
| hsa-miR-497-5p | ACAAACCACAGTGTGCTGCTG |
| U6-F | CGGCAGCACATATAC |
| U6-R | TTCACGAATTTGCGTGTCAT |
| GAPDH-F | TGCCTCCTGCACCACCAACT |
| GAPDH-R | CCCGTTCAGCTCAGGGATGA |
| circRTN4-Over-F | GGTGGTGATATCTTGTTGACCTCCTGTACTGGAGAGACAT |
| circRTN4-Over-R | GGTGGTCCGCGGCTTCAGAGAATCAACTAAATCATCAACT |
| circRTN4-mut-F | GTTTGGTGCCAGCCTATTCCACGAGCTTTCATTGACAGTATTC |
| circRTN4-mut-R | GAATACTGTCAATGAAAGCTCGTGGAATAGGCTGGCACCAAAC |
| sh-cirRTN4-F | GATCTGATTCTCTGAAGTTGTTGATCAAGAGTCAACAACTTCAGAG  AATCTTTTTTG |
| sh-cirRTN4-R | AATTCAAAAAAGATTCTCTGAAGTTGTTGACTCTTGATCAACAACT  TCAGAGAATCA |

**Supplementary Table 3. Coding potential analysis of circRTN4 by Coding Potential Assessment Tool.**

| CPAT: Coding-Potential Assessment Tool | | | | | | |
| --- | --- | --- | --- | --- | --- | --- |
| Sequence Name | RNA Size | ORF Size | Ficket Score | Hexamer Score | Coding Probability | Coding Label |
| circRTN4 | 347 | 96 | 1.1141 | -0.175153881602 | 0.0086970285073155 | no |
